# Editorial: Risk assessment of mycotoxins on metabolism, immunity, and intestinal health

**DOI:** 10.3389/fmicb.2024.1367261

**Published:** 2024-02-09

**Authors:** Einar Ringø, Xiaoyum He, Xiao Li Shen, Jing Jin, Fuguo Xing

**Affiliations:** ^1^Faculty of Bioscience, Fisheries, and Economics, Norwegian College of Fishery Science, UiT The Arctic University of Norway, Tromsø, Norway; ^2^College of Food Science and Nutritional Engineering, China Agricultural University, Beijing, China; ^3^School of Public Health, Zunyi Medical University, Zunyi, Guizhou, China; ^4^Institute of Food Science and Technology, Chinese Academy of Agricultural Sciences, Beijing, China

**Keywords:** mycotoxins, metabolism, immunity, intestinal health, detoxification

Mycotoxins are toxic secondary metabolites produced by mycotoxigenic filamentous fungi such as *Aspergillus, Alternaria, Fusarium*, and *Penicillium*. More than 400 mycotoxins are identified (Ji et al., 2016), and they can contaminate a wide variety of foods (e.g., Pereira et al., 2014; Chen et al., 2018; Jin et al., 2021, 2022; Deng et al., 2023; Hamad et al., 2023; Zhang et al., 2023) as well as fish feed (e.g., Goncalves et al., 2018; Oliveira and Vasconcelos, 2020). The topic of mycotoxins is of significant concern, and according to the Web of Science, using the keyword mycotoxins, approximately 22,000 papers are published *per se*, and how mycotoxins affect host health is important.

Mycotoxins cause growth depression and reduced fertility, feed intake, nutrient utilization, reproduction, and egg production and hatchability as well as an endocrine effect on humans and on pregnancy. In addition, mycotoxins affect metabolism, suppress the immune system, manifesting as depressed T- or B-lymphocyte activity, suppress antibody production, impair macrophage/neutrophil-effector functions, contribute to vaccine failure, impair gut health, and affect intestinal microbiota and the effects of probiotics on detoxification of mycotoxins *in vivo* and *in vitro*.

The connections between gut microbiota and the gut-brain axis are a hot topic; however, the connection between mycotoxins and the gut-brain axis is, to the best of our knowledge, less investigated (Wu et al., 2023), and whether mycotoxins may affect the signaling pathways is an open question. Furthermore, in a recent review by Amorim Neto and Sant'ana (2023), the authors discussed the mycobiota and gut-brain axis in Parkinson's disease (PD), and suggested that mold and their metabolites could induce clinical PD symptoms.

One study in the Research Topic “*Risk assessment of mycotoxins on metabolism, immunity, and intestinal health*” investigated Brevin-1 isolated from Chinese edible frog (*Hoplobatravhus rugulosus*) and revealed LPS-neutralizing and anti-inflammatory activities *in vitro* and *in vivo*. Kumari and Singh evaluated the effect of cinnamaldehyde, an essential oil in the bark of cinnamon trees, on the histopathology and biological parameters in different organs of Swiss albino mice; the results indicated the protective role of cinnamaldehyde against tenuazonic acid isolated from marine fungi (*Paradendryphiella arenariae*). Yin et al. revealed the different dose-response effects on the histopathology of haemocytes in muscle cells, body fat, and Malpighian tubules of domestic silkworm (*Bombyx mori*) by Destruxin A, a mycotoxin from the fungus (*Metarhizium anisopliae*). Even though the study of Qin et al. revealed some uncertainties, the study estimated that hepatocellular carcinoma, through dietary intake of three foods contaminated with aflatoxin in the population of a mountain city in southwest China, was relatively low. However, as no evaluations of the effect of aflatoxin on larger populations are available, the topic merits investigations.

In a study investigating the inhibitory effect of quercetin, a well-known flavonoid, toward *Alicyclobacillus acidoterrestris*, a Gram-positive bacterium, and scanning electron evaluations showed that quercetin could induce irreversible damage of the *A. acidoterrestris* cell membrane and could enhance the surface hydrophobicity of the bacteria. The paper by Zou et al. discussed the characteristics and effectiveness of traditional Chinese medicine and their mechanisms of action: antiviral (active components such as alkaloids, flavonoids, saponins, quinones etc.,), antimicrobial (flavonoids and flavonoid glycosides), induction of apoptosis, immunomodulatory mechanisms via modulation of immune cell activity, the enhancement of the function of lymphocytes, macrophages, and natural killer cells, and antioxidant mechanism.

In a simulation study on experimental mice, the authors revealed that after ozone degradation, the toxicity of corn contaminated with degrading deoxynivalenol (DON) was significantly lower, with a significant decrease in DON. Based on their results, the authors suggested that the mouse model could be used as an approach to evaluate the effect of fungal toxins on metabolism, immunity, and gut health.

These studies provided interesting results for the scientific and global community, and to conclude, the studies in this Research Topic highlighted the importance of additional studies on mycotoxins. Future studies should focus on how mycotoxins affect physiology, nutrition, homeostasis, gut health, the gut-brain axis, and disease resistance. In addition, the use of non-aflatoxigenic *Aspergillus flavus* as a biocontrol merits further investigation, even though numerous studies were cited in the comprehensive review of More (2022). The rapid development of nanotechnology has revealed several opportunities for determining mycotoxins in foods, even at minute concentrations (Yang et al., 2020); the use of nanotechnology in combination with probiotic delivery for pretreatment might be an option for future studies. Even though our knowledge on mycotoxins has increased significantly since the first studies on mycotoxins were published in the early 1960s (Schumater et al., 1961; Armbrecht et al., 1963), the effect of mycotoxins on metabolism, immunity, and intestinal health still merits further investigation.

## Author contributions

ER: Writing – original draft. XH: Writing – review & editing. XS: Writing – review & editing. JJ: Writing – review & editing. FX: Writing – review & editing.
